# Single Molecule Imaging of T-DNA Intermediates Following *Agrobacterium tumefaciens* Infection in *Nicotiana benthamiana*

**DOI:** 10.3390/ijms20246209

**Published:** 2019-12-09

**Authors:** Idan Pereman, Cathy Melamed-Bessudo, Tal Dahan-Meir, Elad Herz, Michael Elbaum, Avraham A. Levy

**Affiliations:** 1 Department of Plant and Environmental Sciences, The Weizmann Institute of Science, Rehovot 76100, Israel; 2 Migal, Galilee Research Institute, Kiryat Shmona 11016, Israel; 3 Department of Chemical and Biological Physics, The Weizmann Institute of Science, Rehovot 76100, Israel

**Keywords:** T-DNA, live imaging, agrobacterium, *Nicotiana benthamiana*

## Abstract

Plant transformation mediated by *Agrobacterium tumefaciens* is a well-studied phenomenon in which a bacterial DNA fragment (T-DNA), is transferred to the host plant cell, as a single strand, via type IV secretion system and has the potential to reach the nucleus and to be integrated into its genome. While Agrobacterium-mediated transformation has been widely used for laboratory-research and in breeding, the time-course of its journey from the bacterium to the nucleus, the conversion from single- to double-strand intermediates and several aspects of the integration in the genome remain obscure. In this study, we sought to follow T-DNA infection directly using single-molecule live imaging. To this end, we applied the LacO-LacI imaging system in *Nicotiana benthamiana,* which enabled us to identify double-stranded T-DNA (dsT-DNA) molecules as fluorescent foci. Using confocal microscopy, we detected progressive accumulation of dsT-DNA foci in the nucleus, starting 23 h after transfection and reaching an average of 5.4 and 8 foci per nucleus at 48 and 72 h post-infection, respectively. A time-course diffusion analysis of the T-DNA foci has demonstrated their spatial confinement.

## 1. Introduction

*Agrobacterium tumefaciens* (*A. tumefaciens*) is a Gram-negative pathogen that causes crown gall-tumors in dicotyledonous plants [1]. The first indications for its role in the formation of the crown gall date back to the beginning of the 20th century [2] and its development as a tool for genetic transformation of plants was reported in the early 1980s [3]. Since then, *A. tumefaciens* became the primary vector for producing transgenic plants in agronomic and horticultural species. The transfer of the bacteria’s DNA to the plant cell nucleus is mediated by a virulence-induced type IV secretion system in which a tumor-inducing (Ti) plasmid harbors the virulence genes [4]. Several plant genes were found to facilitate T-DNA integration [5,6,7] and more recently, polymerase-theta was shown to be an essential factor for T-DNA integration [8]. Studies which focused on the initial stages of infection and that were set to evaluate the time-course from infection to integration, have detected T-DNA transcription 18 h post infection [9]. Later on, high-throughput analysis of T-DNA-host genome junctions could detect newly formed junctions as soon as 6 h post-infection of Arabidopsis seedlings’ roots [10]. T-DNA molecules end up integrating into the genome, sometimes in tandem or inverted repeat configuration [11]. Whether the T-DNA is integrated as a single-strand T-DNA (ssT-DNA) or as a double-strand T-DNA (dsT-DNA) remains an open question and there is evidence in support of both pathways [12,13,14]. Initial studies of Agrobacterium-mediated transformation in tobacco protoplasts have indicated that the cells are equally competent and that a single or few bacteria transform each cell [15]. Following, a high percentage (18%) of Agrobacterium-mediated transformed protoplasts was observed, alongside with a demonstration of the expression stability of integrated T-DNA molecules in the regenerated plants [16]. A more recent analysis of the number of integrated copies following floral dipping-mediated transformation in Arabidopsis has resulted in an average number of 4–6 T-DNA copies, some of which exhibiting the complexity of tandem and inverted repeats at the integration locus, leading to the suggestion that replication of the T-DNA might precede its final integration [17,18]. Alternatively, co-integration of extrachromosomal T-DNA molecules could give rise to a multi-copy insert [19]. In turn, the integration pattern can affect the stability and expression level of the transgene and this is of concern in agricultural and bio-industrial applications [18,20]. While the transient nature of T-DNA expression following transformation was addressed before [13,21,22], the corresponding number of unintegrated T-DNA molecules could not be directly determined, mainly due to their temporal instability and numerical heterogeneity among individual cells. A plausible approach to resolve this complication could be the direct imaging of the T-DNA molecules throughout the steps of transfection. So far, analysis of T-DNA transfection dynamics focused mainly on the ssT-DNA molecule in its initial steps (see [23,24] for review). Due to shortcomings of available imaging methods to detect single-strand DNA molecules in vivo, data regarding ssT-DNA dynamics were gained indirectly through the study of the structure and the mobility of the VirE2 protein [25,26,27]. The latter imaging studies have provided a putative description of ssT-DNA molecule trafficking in the cytoplasm, thus complementing the molecular observations which have indicated ssT-DNA conversion to dsT-DNA in the nucleus [28]. However, there is currently no data regarding the sequential diffusion dynamics of the dsT-DNA molecules in the nucleoplasm during the final steps of Agrobacteria transfection.

In this study, we used single-molecule live-imaging of T-DNA seen as fluorescent foci, using the LacO-LacI system [29,30,31] that enables to monitor and to track dsT-DNA molecules. Methodologically, this is achieved through the binding of a fluorescent LacI protein fused to a fluorescent mRFP protein to the lacO motif which is present in multiple copies on the dsT-DNA molecule. We addressed the time-course from infection of *Nicotiana benthamiana* (*N. benthamiana*) leaves until the formation of a double-stranded molecule, the copy number of dsT-DNA molecules in a single nucleus and the mobility of the T-DNA. Our main findings are that dsT-DNA molecules can be detected 23 h after transfection in the nucleus and continue to accumulate throughout the following 48 and 72 h, reaching an average of 8 foci per nucleus. The foci of the T-DNA molecules appeared to be stationary and could correspond to extrachromosomal or integrated molecules.

## 2. Results

### 2.1. Visualization of LacO-Array Plasmids in Protoplasts Following Polyethylene Glycol (PEG) Mediated Transformation

**Figure 1:**
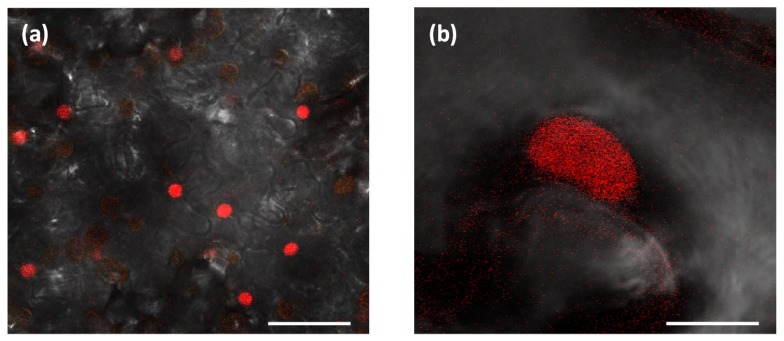
Homogeneous distribution of mRFP-LacI in a transgenic *N. benthamiana* line. The mRFP-LacI protein is homogeneously distributed in the nuclei. (**a**) Scale bar: 50 µm; (**b**) scale bar: 10 µm.

In order to test whether the LacO-LacI imaging system could be applied to detect transient double-strand DNA molecules in *N. benthamiana* cells, we transformed *N. benthamiana* plants with plasmid pCambia 15mRL (Supplemental Figure S1a) which stably expresses an NLS-LacI protein fused to a mRFP fluorescent marker and selected for plants showing a uniform distribution of the mRFP-LacI fluorescent protein in the nucleus (Figure 1). No foci were seen in these plants as expected from the use of the monomeric RFP that does not form aggregates.

**Figure 2:**
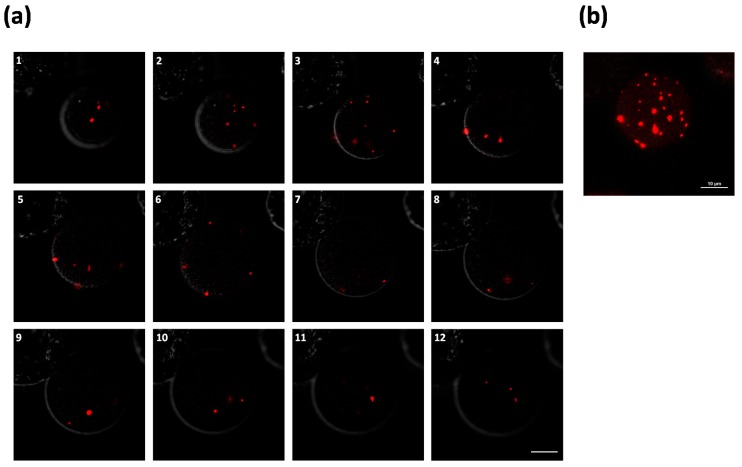
Plasmid DNA detection with the LacO-LacI system in *N. benthamiana* protoplasts. Protoplasts were isolated from mRFP-LacI expressing *N. benthamiana* seedlings. Following PEG transformation with the LacO ×256 double-stranded plasmid p2alpha1 LacO (Supplemental Figure S1b), the protoplasts were imaged 20 h after transformation. (**a**) Stacks of a single protoplast at 2.4 µm intervals.Scale bar: 10 µm; (**b**) Maximum projection of twelve Z-stacks (a1–a12) showing a total of ~30 foci. Scale bar: 10 µm.

As a positive control showing that dsDNA molecules can be detected as foci, we directly transformed protoplasts originating from the transgenic mRFP-LacI plants with a double-stranded plasmid which harbors the LacO array. Foci are expected to form due to the large number of repeats (256 copies) that are bound by the mRFP-LacI protein and each focus is thought to represent a single DNA molecule [31]. Therefore, protoplasts were generated from mRFP-LacI plants followed by PEG-mediated transformation using plasmid p2alpha1 LacO (Supplemental Figure S1b). Indeed, the delivered plasmids were already observed in multiple copies 16 h post transformation (Supplemental Figure S2b) and as much as 30 foci could be observed after 20 h (Figure 2). In a negative control assay, no foci were detected in protoplasts transformed with plasmids lacking the LacO array (Supplemental Figure S2a). Of note, the double-strand plasmids were readily available for LacI-mRFP binding upon entry into the protoplast leading to their detection both in the cytoplasm and nucleus. This shows that dsDNA molecules, when present, can be detected as foci in both the cytoplasm and the nucleus of *N. benthamiana* cells a few hours after transformation.

### 2.2. Detection of dsT-DNA Molecules in N. benthamiana Nuclei Following Agro-Infection

In order to follow the time-course of *A. tumefaciens*-mediated transformation, we monitored the initial time-point at which the first dsT-DNA molecules could be detected inside the host’s nucleus, corresponding to the timing when the ssT-DNA is converted to a dsT-DNA molecule. To this end, the GV3101 *A. tumefaciens* strain containing a Ti-plasmid with the LacO array was infiltrated into leaves of *N. benthamiana* stably expressing the mRFP-LacI in the nucleus. To facilitate detection of transformed cells while enabling efficient screening of hundreds of cells at low magnification (20× objective), a constitutively expressed fluorescent marker (eYFP- driven by a double 35S promoter), was introduced to the 3′ end of the LacO array forming the plasmid p2omega1 LacO eYFP (Supplemental Figure S1c). An initial signal of the eYFP protein could be observed in some of the cells 23 h post infiltration. The nuclei of these cells were further examined at a higher magnification (60× objective) for the presence of red-fluorescent foci. Interestingly, at this time point, transformed cells did not show more than a single focus (a single T-DNA molecule) and T-DNA foci were not detected prior to the eYFP signal detection.

### 2.3. T-DNA Molecules Accumulation in the Nucleus

**Figure 3:**
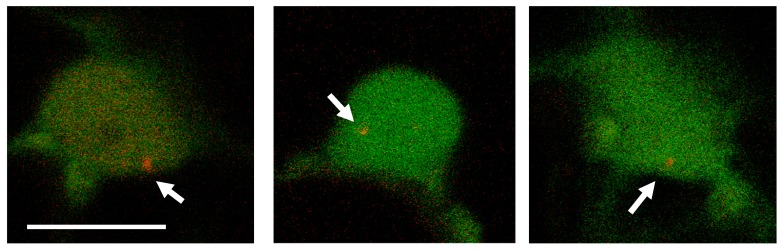
T-DNA molecule detection in *N. benthamiana* nuclei, 23 h following Agrobacterium infection. The GV3101 *A. tumefaciens* strain containing a Ti-plasmid with the LacO eYFP T-DNA was infiltrated into leaves of *N. benthamiana* stably expressing the mRFP-LacI in the nucleus. Foci are shown in three independent nuclei 23 h following transformation. White arrows indicate T-DNA foci. Scale bar: 10 µm.

**Figure 4:**
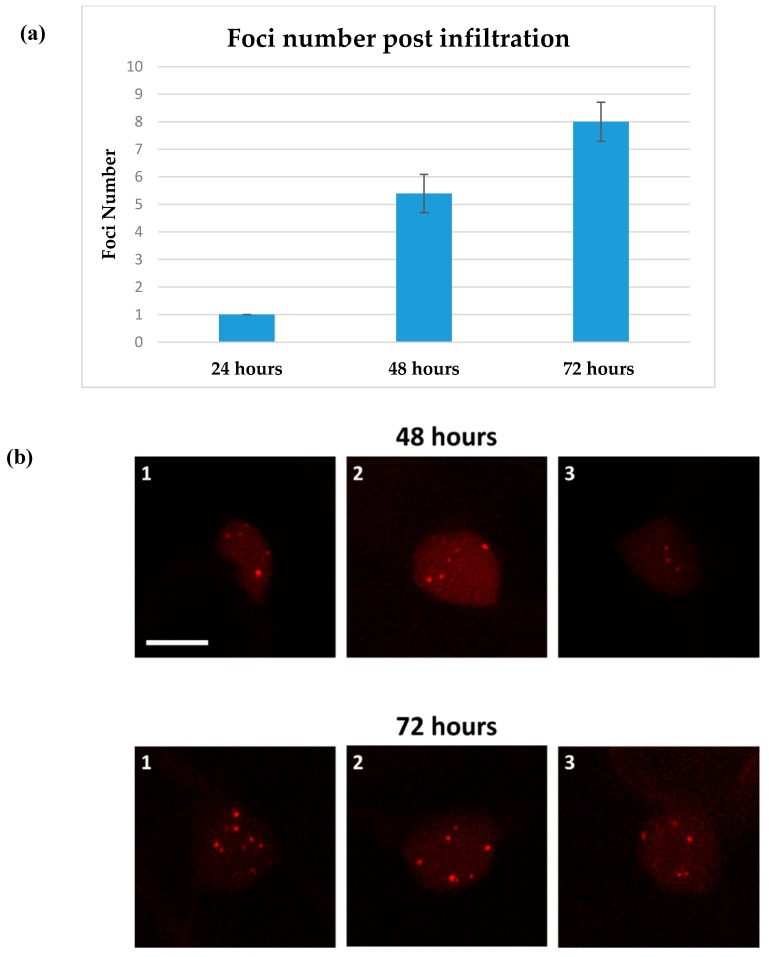
Time-course of T-DNA foci detection in *N. benthamiana*. Leaves stably expressing the mRFP-LacI protein were infected via infiltration using Agrobacterium strain GV3101 containing a Ti-plasmid with the LacO eYFP T-DNA. (**a**) First detection of foci in singular cells 24 h post infection and foci average in 20 nuclei 48 and 72 h post infiltration. Data represent means ± SE. Values show significant difference at *p* < 0.02 according to *t*-Test; (**b**) max projection confocal image of three independent nuclei. Scale bar: 10 µm.

To assess the accumulation of dsT-DNA molecules following their initial detection (at 23 h), leaves infiltrated with the same strains and constructs as used in Figure 3, were analyzed 48 and 72 h post-infiltration and the average number of fluorescent foci was measured in 20 independent cells originating from the same leaf for each time point (Figure 4). This analysis showed progressive accumulation of foci in the nucleoplasm, reaching an average of 5.4 and 8 foci per nucleus at 48 and 72 h post infection respectively. In a negative control assay, no foci could be detected in leaves infiltrated with a construct lacking the LacO array (Supplemental Figure S3).

### 2.4. dsT-DNA Foci Diffusion is Spatially Confined to a Sub-Fraction of the Nuclear Sphere

In order to monitor nuclear localization and movement of a dsT-DNA molecule within the nucleus, we imaged and measured the change over time between a pair of randomly selected foci using the mean squared change (MSC) formula [32]. This has allowed measuring the dsT-DNA diffusion coefficient by monitoring the dynamics of two T-DNA molecules relative to one another and was performed in 10 independent nuclei 72 h following transformation along an interval of 5 min.

(1) $$\mathrm{MSC}=\Delta {d_{1,2}}^{2}={\left\{d_{1,2}\left(t\right)-d_{1,2}\left(t+\Delta t\right)\right\}}^{2}$$

where $d_{1,2}$ is the distance between foci 1 and 2 and Δt = 5 min.

**Figure 5:**
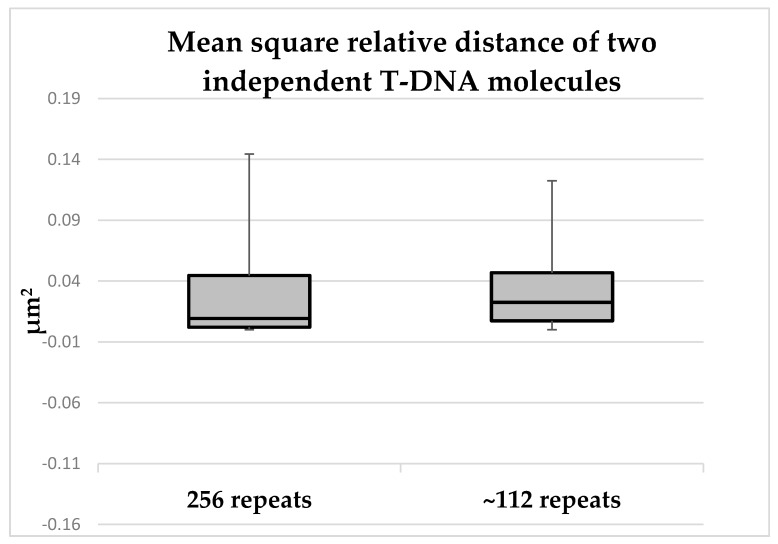
Mean squared change of T-DNA molecules pairs (Boxplot). The mean squared change (MSC) between two independent T-DNA foci was measured in 10 independent nuclei following infiltration with a full LacO array (256 repeats) and a truncated LacO array (~112 repeats). Whiskers represent minimal-maximal values. Difference between MSC values is statistically not-significant according to *t*-Test.

Interestingly, this analysis has measured a maximum distance change between two independent foci of 0.15 µm^2^. This value and its order of magnitude correlate with the diffusion coefficient values which were previously obtained for dense chromatin regions [33]. Moreover, this result also stands in line with works that have applied the LacO-LacI imaging system in yeast cells and demonstrated spatial confinement of plasmid chromatin [32,34]. Next, in order to test a possible correlation between the length of the T-DNA molecule and its mobility, the same analysis was repeated, only this time with a shorter LacO array with an estimate of ~112 repeats. This analysis resulted in similar MSC values, thus demonstrating that the relative diffusion of the T-DNA molecule did not correlate with its length (Figure 5) and corresponds to that expected for stationary molecules.

## 3. Discussion

The imaging system described in this study has provided new insight into some basic aspects of Agrobacterium-mediated infection. The lack of foci in the negative control expressing the mRFP-LacI protein and the presence of foci when using direct PEG transformation with the dsT-DNA plasmid indicate that we can accurately image dsT-DNA molecules. This is further supported by the absence of foci in the cytoplasm upon Agrobacterium-mediated infection, as expected from earlier findings showing that the ssT-DNA is converted into a dsT-DNA within the nucleus. It took 23 h to detect the first evidence of dsT-DNA molecules upon agro-infection (Figure 3). By comparison, detection of T-DNA LacO plasmids, directly transformed into protoplasts, was faster (Figure S2b and Figure 2). This time discrepancy combines the time of infection, the time to reach the nucleus and the conversion from ssT-DNA to dsT-DNA within the nucleus. This 23 h period is much longer that the 6 h reported to detect integration events in Arabidopsis [10]. This might be due to differences between tissues, namely leaf infiltration used here versus root infection in Arabidopsis [10] and/or to differences between species, namely *N. benthamiana* versus *Arabidopsis thaliana*. Interestingly, previous imaging studies of the VirE2 protein have suggested that the time which is required for signal detection correlates with the Vir proteins’ induction time-course and the fluorescent-tagged protein accumulation lag [27,35]. Note that timing of foci detection probably lags behind the actual dsT-DNA transformation kinetics due to the time required for the accumulation and binding of the mRFP-LacI molecules to the lacO×256 array. To date, there was no good estimate for the number of ssT-DNA or dsT-DNA copies in the nucleus. A new insight from this work is the number of 4–8 dsT-DNA-corresponding foci which accumulate gradually 48–72 h post infection. Foci accumulation over time could be due to continuous infection of a given cell by a single bacterium, or from the cumulative effect of independent infections by different bacteria. Typical transformation protocols in tobacco include a 48–72 h typical co-cultivation time [36] and eventually the majority of transgenic plants contain 1–2 T-DNA copies [37]. Assuming that the dsT-DNA are the intermediates of integration, it suggests that only a fraction of the dsT-DNA copies which end up in the nucleus integrate the genome. Furthermore, when taking into consideration the fact that this imaging system does not differentiate between integrated and non-integrated dsT-DNA foci, it also suggests that part of the (maybe all) observed foci represent non-integrated T-DNA molecules. This is consistent with the works of Janssen et al. and De Buck et al. who determined dominancy of transient T-DNA molecules in the initial stages of protein expression [21,38]. Another puzzling observation made here is that, contrary to our initial expectation to identify mobile dsT-DNA intermediates, the foci that were observed exhibited constrained diffusion dynamics. These stationary foci could indicate an initial interaction of the dsT-DNA with a nuclear chromatin region. This could be typical of all dsT-DNA molecules, or alternatively, this might be caused by heterochromatinization of the repetitive array of LacO copies. The lack of mobility was still observed when reducing copy numbers from 256 to 112—yet we cannot rule out that this 112-copies repetitive array is undergoing heterochromatinization. Future works should combine ssT-DNA and dsT-DNA single-molecule imaging with systems that enable more sensitive detection of foci to reduce the need for multi-copy repeat arrays. This should provide a better understanding as to the time-course of conversion of ss to dsT-DNA within the nucleus, intermediate mobility in the nucleus and mechanisms of integration.

## 4. Materials and Methods

### 4.1. Plant Culture

*N. benthamiana* plants were soil-grown in climate rooms (22 °C; 70% humidity; 18: 6 h, light: dark). Leaves of one-month-old plants with an estimated diameter size of 3 cm were used for infiltration.

### 4.2. Microscopy

Nikon Eclipse Ti-E microscope (Tokyo, Japan) with Nikon A1+ confocal laser system equipped with CFI PLAN APO VC × 60 A WI. mRFP detection–excitation: 561 nm emission: 570–620 nm. eYFP detection–excitation: 488 nm emission: 500–550 nm.

### 4.3. Plasmid Construction

The GoldenBraid cloning system (https://gbcloning.upv.es/) was applied for plasmid cloning.

-pDGB2_alpha1_LacO×256: The LacO×256 array was excised from the pAFS59 [39] plasmid using BamHI and HindIII and inserted into pDGB2_alpha1 using the same restriction sites.
-pDGB2_alpha2_eYFP: An eYFP under a double 35S promoter, was amplified from pSAT6 eYFP-N1 using primers 5′-GCGCCGTCTCGCTCGGGAGGGCGAAAGGGGGATGTGCTGC-3′ eYFP Fw, 5′-GCGCCGTCTCGCTCGAGCGCTGGAAAGCGGGCAGTGAGCG-3′ eYFP Rv, restricted with BsmBI and ligated to pUPD. Following, eYFP was restricted from pUPD using BsaI and sub-cloned into pDGB2_alpha2.
-pDGB2_omega1_LacO×256_eYFP: pDGB2_alpha1_LacO×256 and pDGB2_alpha2_eYFP were restricted with BsmBI and ligated into pDGB2_omega1.
-pCambia mRFP: The 35S and NLS-LacI were amplified as two independent fragments from pBC-35S-RL (derived from construct 16, Matzke et al. [29]). An mRFP gene was introduced between the promoter and the NLS-LacI gene by PCR. The complete fragment was ligated to pCAMBIA between EcoRI and HindIII sites.

### 4.4. N. benthamiana Transgenic Line Generation

Transformation was done using Agrobacterium strain GV3101, as described in Clemente, 2006 [36] with modifications. Young leaves of *N. benthamiana* (4–6 cm diameter) were sterilized using 1% Sodium hypochlorite for 10 min, followed by rinsing five times with sterile distilled water. Leaves were cut to 2 cm squares on Miracloth soaked with Agrobacterium (OD600 = 0.4, Murashige & Skoog M0256, Sucrose 3%, pH = 5.2) and transferred to co-cultivation plates (Murashige & Skoog M0222, Sucrose 3%, Agar 0.9%, Acetosyringone 100 uM, with a single piece of sterile Whatman filter paper) for 48 h at 22 °C at dark. Following co-cultivation, cut leaves were transferred to 1st selection regeneration medium (Murashige & Skoog M0231, Sucrose 3%, Agar 0.9%, Ticarcillin 400 mg/L, Kanamycin 100 mg/L, IAA 0.8 mg/L, Kinetin 2 mg/L) for two weeks, then moved to 2nd selection regeneration medium (Murashige & Skoog M0231, Sucrose 3%, Agar 0.9%, Ticarcillin 200 mg/L, Kanamycin 100 mg/L, IAA 0.8 mg/L, Kinetin 2 mg/L). Plantlets were transferred to rooting medium (Murashige & Skoog M0231, Sucrose 3%, Agar 0.9%, Ticarcillin 100 mg/L, Kanamycin 500 mg/L, IBA 1 mg/L) for two weeks. Rooted plantlets were moved to soil.

### 4.5. Agro-Infiltration in N. benthamiana

Following electro-transformation with 1µl DNA plasmid, agrobacterium GV3101(pMP90) was spread on LB plates containing the appropriate antibiotics (Rifampicin 20 mg/L, Gentamicin 50 mg/L, spectinomycin 100 mg/L) and incubated for 2 days at 28 °C. The bacteria were then scraped using a rubber policeman, washed twice with a solution containing 10 mM MgCl2, 1 µm acetosyringone, and diluted with the same solution to OD600 = 0.5. The bacteria-suspension was infiltrated with a 1 mL syringe into young leaves.

### 4.6. Protoplast Isolation and PEG-Mediated Transformation

Protoplasts were isolated and transformed as described in Yoo et al. [40] with modifications: tobacco seeds were sown in 10 cm petri dishes and were grown for 8–16 h at 25 °C for three weeks. The seedlings were harvested and incubated in “enzyme solution” for 4 h. For the transformation, 2 µg of plasmid (in 10 µL) were used.

## Supplementary Materials

Supplementary materials can be found at https://www.mdpi.com/1422-0067/20/24/6209/s1.
