# Supplementary material for: Single Molecule Imaging of T-DNA Intermediates Following Agrobacterium tumefaciens Infection in Nicotiana benthamiana
Source: Int J Mol Sci. 2019 Dec 9;20(24):6209. doi: 10.3390/ijms20246209 (PMC6940882; doi:10.3390/ijms20246209)
Supplement: Supplementary file 1 [file ijms-20-06209-s001.pdf]

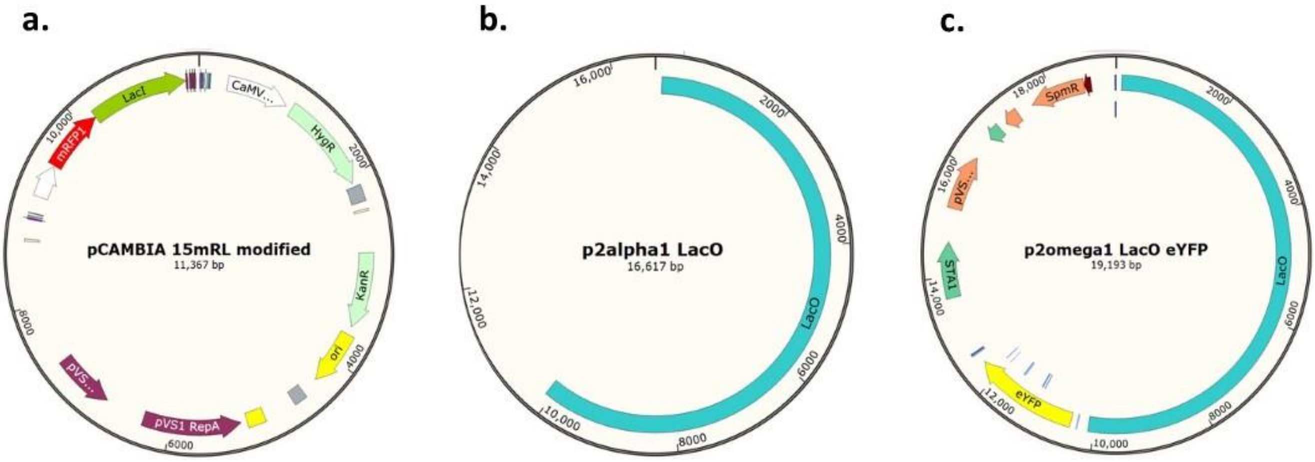


**Supplemental figure S1. Plasmids used in the study for dsT-DNA imaging.**
(a) pCambia 15mRL (b) p2alpha1 LacO (c) p2omega1 LacO eYFP.

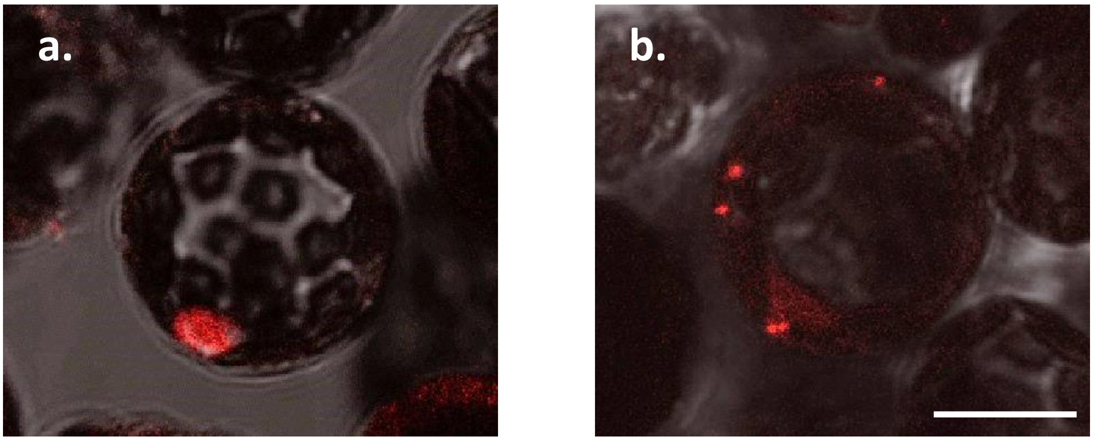


**Supplemental figure S2. Plasmid DNA detection with the LacO-LacI system in *N. benthamiana* protoplasts.** Protoplasts were isolated from mRFP-LacI expressing *N. benthamiana* seedlings, subjected to PEG transformation and imaged 16 hours after transformation.(**a**) A single protoplast transformed with a double-stranded control plasmid lacking the lacO array ; (**b** A single protoplast transformed with the LacOx256 double-stranded plasmid p2alpha1 LacO (Supplemental image S1b), Scale bar: 10μm.

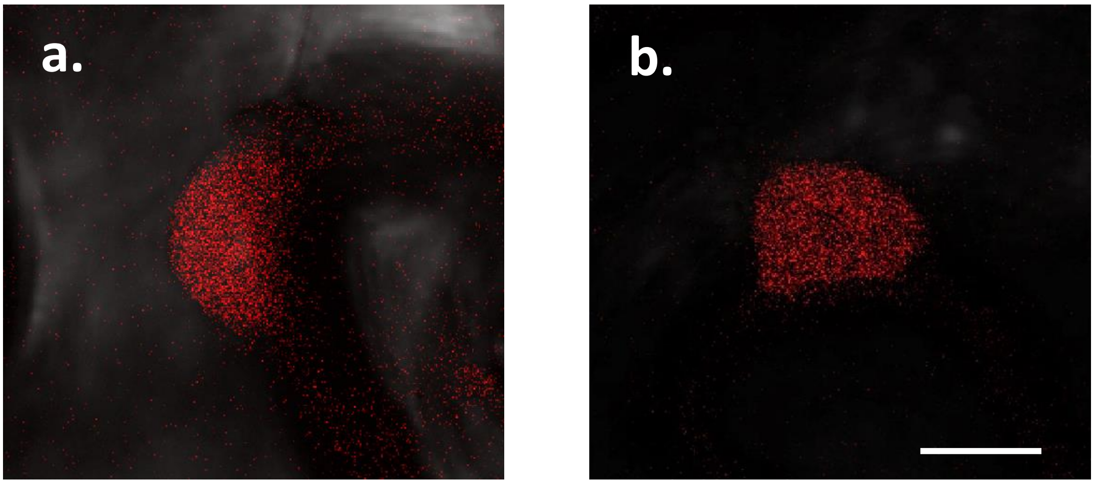


**Supplemental figure S3. Time course of T-DNA foci detection in *N. benthamiana*.** Leaves stably expressing the mRFP-LacI protein were infected via infiltration using Agrobacterium strain GV3101 containing a control Ti-plasmid lacking the LacO array. (a) 50 hours post infiltration; (b) 72 hours post infiltration. Scale bar: 10μm.
